# The Anti-Tuberculosis Campaign

**Published:** 1920-11-27

**Authors:** 


### THE ANTI-TUBERCULOSIS CAMPAIGN.

**By a Correspondent.**

The current time-spirit of flux and uncertainty manifests itself even in our anti-tuberculosis activities. The end of the main war in which this country was involved set free for civil and municipal work a good number of medical men, while certain revelations of this country's backwardness, made in the earlier part of the contest, when her cause looked unhopeful, lingered in their minds. On top of these things came a strong, and in certain quarters unlooked-for, upgrowth of Labour and Socialist sympathy, naturally favouring as much public-health work as possible. Result, a stirring of the waters of sanitary effort, although a stirring rather superficial. There were secondary causes, too. The huge increase in tuberculosis that the war brought to every European State that mobilised necessitated hurried, slipshod treatment of the disease. Sent to a hutment sanatorium, under the orders of a nursing staff, and with only a short daily visit from a medical man, the consumptive ex-soldier, suffering also from another consequence of war—namely, demoralisation—got drunk, or stopped out all night, or simply took his departure for more congenial environment, with an astonishing frequency. It would be safe to say that not a single public sanatorium kept 60 per cent. of its patients for a full term of treatment, or is now keeping them. This is one of the two great points tuberculosis workers interested in sanatoriums should consider, the other being the development of their institutional grounds, and their institutional domestic economy, in the direction of providing suitable employment for ex-patients, since the demand for such exceeds the supply a hundredfold.

### Professor Hill's Work.

Now, Professor Hill has of course argued in a very interesting way that the good effects of open air are due not so much, if at all, to the relative absence of carbonic acid and of organic pollution as to stimulative action of air currents and of cold upon the cutaneous nerves. But open air is implicit in sanatorium treatment, and has always been so. When British workers took up the hygienic-dietetic treatment of tuberculosis, if anything they rather overdid the "let him have air, he'll straight be well" component. None more than this journal believes in scientific discussion. But at a time like the present, when faults already indicated, and others, like insecurity of tenure of superintendentship, insufficient authority, lack of a high clinical standard, and so forth, loom so large, it would have been better for a new-formed society to attack them direct.

The Leeds Conference was on more practical lines, although, for some reason, the actual rally of speakers was not so representative as the first announcement of names. Sir Robert Philip's recent address in Edinburgh sounded, to tell the truth, a little behind the times. What is now needed is willing facing of the fact that the contagionist doctrine of tuberculosis in vogue pre-war will not do any longer. War tuberculosis was not due to infection; it came far too quickly, far too widely, far too independently of existing cases of the disease. It was due to depressed constitutional resistance setting free, making active and progressive, slight lesions which an almost ubiquitous bacillus causes in the great majority of a civilised community. And thus prophylaxis connotes, to a hitherto unsuspectedly high degree, the raising and maintaining of individual constitutional resistance. The means of this? Surely economic. Tubercle is rare, as a general thing, in the middle class. Secure, therefore, middle-class conditions of life for all, and you decimate tubercle. How glibly in the old days we used to say that tuberculosis was a medico-sociological problem, yet how we failed to realise this! At the same meeting Sir Henry Gauvain said things he has probably often said before, but probably not yet said enough. There is a risk of the treatment and diagnosis of surgical tubercle being lumped in with general orthopædics. This would be a great pity on several scores. If we except Mr. Elmslie, perhaps no general orthopædist knows so well how to treat surgical tubercle as a specialist in that disease. Secondly, no general orthopædist is going to live with his hospital cases and be responsible for their daily progress, which is what is needed for efficiency; he will rely on frequently changing house surgeons, and treatment will suffer. Thirdly, tuberculous children are surely best segregated.

### United Efforts.

If we come to less urgent matters, the incorporation of tuberculosis workers, now pretty complete all over the country, is certainly to the good. Years ago German tuberculosis specialists banded themselves into similar unions, which have endured. The specialty has now in the journal *Tubercle* an organ in which are available from the foreign press abstracts of many interesting papers. A greater knowledge of tuberculosis literature current abroad is certainly desirable, if only for the saving of many a needless paper being written in these days of shortage of everything, most of all of truth. Finally, the Tuberculosis Society has shown a praiseworthy preference for professional over material attainment. It would seem as if the readiest path of advancement for a tuberculosis officer would be to cultivate his administrative knowledge, take up side-lines of sanitary work, and aim at becoming as soon as possible a medical officer of health. But that would not be the way to acquire clinical skill, or, indeed, in the long run perhaps, to better his calling. The maxim "Be good doctors" did wonders for the old R.A.M.C. If the tuberculosis officer seeks first the kingdom of technical efficiency, all the other things may well be added to him.

